# Assessment of Complications and Length of Hospital Stay Associated With Surgical Emergencies in Patients With Concurrent COVID-19 Infection

**DOI:** 10.7759/cureus.68965

**Published:** 2024-09-08

**Authors:** Vaishnavi Kannan, J V Pranav Sharma

**Affiliations:** 1 General Surgery, Vydehi Institute of Medical Sciences and Research Centre, Bangalore, IND; 2 General Surgery, Adesh Medical College and Hospital, Shahbad, IND

**Keywords:** covid-19, emergency surgery, hospital stay, icu admissions, patient recovery, postoperative complications, surgical outcomes

## Abstract

The delivery of surgical services was profoundly affected by the COVID-19 pandemic, resulting in the postponement of elective surgeries and a shift in focus to essential emergency procedures. Our study aimed to assess the impact of concurrent COVID-19 infection on complications, hospital stay, and recovery following emergency surgery. A retrospective matched cohort study was conducted between July 2020 and February 2022 at a tertiary care hospital in India. Data from 48 patients with COVID-19 infection in the immediate preoperative period was compared with 48 matched controls not infected with the virus. The data collected included patient demographics, surgical procedures, duration of hospital stay, and postoperative complications. Patients with concurrent COVID-19 infection had notably longer mean hospital stays (13.44 days) than the controls (6.63 days) (P = 0.002). An elevated proportion of COVID-19-positive patients experienced discharge delays (36 out of 48, 75%), compared to just six of the 48 non-COVID-19 patients (12.5%) (P ≤ 0.001). Postoperative findings in the COVID-positive cohort revealed elevated rates of pulmonary complications (5/48, 10.4%), higher rates of postoperative ICU admissions (8/48, 16.7%), and persistently elevated D-dimer levels extending beyond postoperative day seven (18/48, 37.5%). This suggests that emergency surgery in patients with COVID-19 is linked to significantly lengthier hospital stays, increased discharge delays, and a greater prevalence of adverse events in the postoperative period when compared to controls. These findings underscore the need for enhanced perioperative strategies and preparedness for potential future pandemics.

## Introduction

SARS-CoV-2 infection or COVID-19 is a viral infectious zoonotic disease that primarily targets the respiratory system. Its rapid spread worldwide led to its official recognition as a global pandemic on March 11, 2020, by the World Health Organization [1].

This global health crisis presented substantial challenges for surgeons worldwide, compelling them to balance the need for timely surgical interventions with the risks associated with operating on COVID-19 patients [2]. Consequently, elective surgical cases were largely deferred, with surgeons handling only surgical emergencies and cancer patients requiring surgery [3]. Surgical interventions for COVID-19 patients were carried out with stringent safety measures, including limited staff presence, optimized operating room configurations, and strict compliance with personal protective equipment (PPE) protocols. Concerns regarding viral dissemination from surgical smoke led to the cautious use of aerosol-generating procedures such as laparoscopy and surgeries involving electrocautery, lasers, or ultrasonic scalpels. These precautions were intended to mitigate risks such as potential viral leakage from the pneumoperitoneum and increased smoke production during surgery [4].

Despite these measures, research consistently shows that COVID-19 patients undergoing emergency surgical procedures face significantly extended hospitalizations, increased rates of postoperative complications, and elevated 30-day mortality rates when compared to patients without COVID-19 infection. Furthermore, these patients are more likely to require admission to the intensive care unit (ICU) following surgery. These findings highlight the severe impact of COVID-19 on surgical outcomes and reinforce the need for careful perioperative management in this patient population [2,5-10].

Understanding how SARS-CoV-2 impacts surgical outcomes is critical for enhancing surgical protocols and strategizing surgical services for anticipated pandemics in the future. This study aims to evaluate the surgical outcomes of COVID-19 patients undergoing emergency surgery in comparison to patients without COVID-19 undergoing similar procedures.

## Materials and methods

Study design

This was a retrospective matched cohort study conducted at the Vydehi Institute of Medical Sciences and Research Centre, Bangalore, India, from July 2020 to February 2022 to evaluate surgical outcome metrics in COVID-19 patients undergoing emergency surgical procedures as compared to the matched controls. The data analyzed in this study were collected from patient records. The study was approved by the Vydehi Institutional Ethics Committee (approval number: VIEC/2022/APP/036; EC Reg No: ECR/747/Inst/KA/2015/RR-21) on October 3, 2022. Due to the retrospective nature of the study, informed consent from individual patients was waived.

Population under study

Patients who tested positive for COVID-19 shortly before undergoing emergency surgical procedures were included in the study. These patients were matched with a control group that comprised patients without COVID-19 infection who underwent similar emergency surgery procedures during the same time period. The inclusion and exclusion criteria for this study are presented in Table 1.

**Table 1:** Inclusion and exclusion criteria

Criteria	Inclusion Criteria	Exclusion Criteria
Age	Adult patients (18 years and older)	Patients under 18 years
COVID-19 Status	Tested positive for COVID-19 before and during the surgical intervention	Tested negative for COVID-19 before the surgical intervention
Procedure Type	Emergency surgeries only	Elective or non-emergency surgeries
Time Frame	Procedures conducted between July 2020 and February 2022	Procedures outside this time frame
Medical Records	Complete and accessible medical records for analysis	Incomplete or inaccessible medical records

Data collection

Data for this study were obtained from electronic and paper-based medical records at the hospital, encompassing emergency surgery procedures conducted within the aforementioned time frame. Consent was obtained from the respective department heads to access the medical records of their surgical patients. Relevant data were extracted, including patient demographics, preoperative COVID-19 status, type of surgery, length of hospital stay, intraoperative findings, and postoperative outcomes leading up to their date of discharge. All patient data were anonymized to ensure confidentiality, with access restricted to designated individuals involved in the research study. Data entries were cross-checked with the medical records to maintain consistency and accuracy.

Statistical analysis

Descriptive and inferential statistical analyses were conducted for this study. Continuous variables were summarized as mean ± standard deviation (SD) with the range (minimum-maximum), while categorical variables were presented as frequency (percentage). Statistical significance was assessed at a 5% significance level. Assumptions include normal distribution of dependent variables, random sampling, and independence of observations. For categorical data, the Chi-square test or Fisher’s Exact test was used to determine significance between groups, with Fisher’s Exact test applied specifically when sample sizes in contingency tables were small. Non-parametric methods were used where appropriate for qualitative data analysis. Significance levels were classified as follows: suggestive (0.05 < P ≤ 0.10), moderately significant (0.01 < P ≤ 0.05), and strongly significant (P ≤ 0.01). The statistical analyses were conducted using IBM SPSS Statistics for Windows, Version 22.0 (Released 2013; IBM Corp., Armonk, New York, United States) [11] and R version 3.2.2 (R Foundation for Statistical Computing, Vienna, Austria) [12], while Microsoft Word and Excel (Microsoft Corporation, Redmond, Washington, United States) were used to generate tables.

## Results

Patient demographics and baseline characteristics

Our study included 96 patients, equally divided into two groups: Group 1 (n=48) comprised patients who were COVID-19-positive, and Group 2 (n=48) comprised patients who were COVID-19-negative. Both groups were matched for age, gender, surgical specialty, surgical procedures done, and type of anesthesia administered.

The age distribution of patients is presented in Table 2, showing a balanced match between the two groups across different age categories. The mean age of the COVID-positive group was 36.1 years, while that of the COVID-negative group was 37.15 years.

**Table 2:** Age distribution of patients Samples are age-matched with p-value = 0.767, Student t-test

Age (Years)	COVID-19-Positive (n=48), n (%)	COVID-19-Negative (n=48), n (%)	Total (N=96), n (%)
<20	2 (4.2%)	0 (0%)	2 (2.1%)
20-30	30 (62.5%)	28 (58.3%)	58 (60.4%)
31-40	4 (8.3%)	5 (10.4%)	9 (9.4%)
>40	12 (25%)	15 (31.3%)	27 (28.1%)
Age, mean±SD	36.1±18.59	37.15±15.55	36.63±17.05

The majority of patients in both groups were female, with 34 out of 48 (70.8%) in the COVID-positive group and 33 out of 48 (68.8%) in the COVID-negative group, as outlined in Table 3.

**Table 3:** Gender distribution of patients (N=96) P-value = 1.000, not significant, Chi-Square Test

Gender	COVID-19-Positive (n=48), n (%)	COVID-19-Negative (n=48), n (%)	Total (n=96), n (%)
Female	34 (70.8%)	33 (68.8%)	67 (69.8%)
Male	14 (29.2%)	15 (31.3%)	29 (30.2%)

In our study, Obstetrics and Gynecology emerged as the most frequently encountered surgical specialty for emergency procedures, with 31 cases (64.6%) in both the COVID-19-positive and COVID-19-negative groups. This distribution is detailed in Table 4, which summarizes the surgical specialties involved in the study.

**Table 4:** Distribution of patients according to surgical specialty Chi-Square Test/Fisher Exact Test

Surgical Specialty	COVID-19-Positive (n=48), n (%)	COVID-19-Negative (n=48), n (%)	Total (n=96), n (%)	p-value
Obstetrics and Gynecology	31 (64.6%)	31 (64.6%)	62 (64.6%)	0.823
Otolaryngology	7 (14.6%)	7 (14.6%)	13 (13.5%)	0.777
General Surgery	7 (14.6%)	7 (14.6%)	15 (15.6%)	0.777
Surgical Oncology	2 (4.2%)	2 (4.2%)	4 (4.2%)	1.000
Orthopedics	1 (2.1%)	1 (2.1%)	2 (2.1%)	1.000
Urology	1 (2.1%)	1 (2.1%)	2 (2.1%)	1.000

Emergency cesarean section was the most commonly performed procedure, with 31 out of 48 cases (64.6%) in both the COVID-19-positive and COVID-19-negative groups. The specific surgical procedures performed are listed in Table 5.

**Table 5:** Distribution of patients according to surgical procedure DJ: double-J, FESS: functional endoscopic sinus surgery

Surgical Procedure	COVID-19-Positive (n=48), n (%)	COVID-19-Negative (n=48), n (%)	Total (n=96), n (%)
Emergency Caesarean Section	31 (64.6%)	31 (64.6%)	62 (64.6%)
Emergency Tracheostomy	3 (6.3%)	3 (6.3%)	6 (6.3%)
Fasciotomy	1 (2.1%)	1 (2.1%)	2 (2.1%)
DJ Stent Removal	1 (2.1%)	1 (2.1%)	2 (2.1%)
FESS with Debridement	3 (6.3%)	3 (6.3%)	6 (6.3%)
Below Knee Amputation	2 (4.2%)	2 (4.2%)	3 (3.1%)
Ray Amputation	2 (4.2%)	2 (4.2%)	4 (4.2%)
Debridement	1 (2.1%)	1 (2.1%)	2 (2.1%)
Colostomy	1 (2.1%)	1 (2.1%)	2 (2.1%)
Emergency Laparotomy	1 (2.1%)	1 (2.1%)	2 (2.1%)
Open Cholecystectomy	1 (2.1%)	1 (2.1%)	2 (2.1%)

Spinal anesthesia was the predominant form of anesthesia administered, used in 35 out of 48 cases (72.9%) in both groups. An overview of the types of anesthesia administered for emergency procedures in the study is provided in Table 6.

**Table 6:** Distribution of patients according to anesthesia administered

Anesthesia Administered	COVID-19-Positive (n=48), n (%)	COVID-19-Negative (n=48), n (%)	Total (n=96), n (%)
Spinal Anesthesia	35 (72.9%)	35 (72.9%)	66 (68.8%)
General Anesthesia	11 (22.9%)	11 (22.9%)	20 (20.8%)
Local Anesthesia	1 (2.9)	1 (2.1%)	3 (3.1%)
Ankle Block	1 (2.1%)	1 (2.1%)	2 (2.1%)

The distribution of comorbidities between the two cohorts shows a slightly higher percentage of comorbid conditions in the COVID-19-negative group (26 out of 48, 54.2%) compared to the COVID-positive group (21 out of 48, 43.8%). The distribution of comorbidities across both groups is detailed in Table 7.

**Table 7:** Distribution of patients according to comorbidities P-value = 0.413, not significant, Chi-Square Test

Comorbities	COVID-19-Positive (n=48), n (%)	COVID-19-Negative (n=48), n (%)	Total (n=96), n (%)
ABSENT	27 (56.3%)	22 (45.8%)	49 (51%)
PRESENT	21 (43.8%)	26 (54.2%)	47 (49%)
Type 2 Diabetes Mellitus	11 (22.9%)	17 (35.4%)	30 (31.3%)
Hypothyroidism	8 (16.7%)	7 (14.6%)	15 (15.6%)
Gestational Diabetes Mellitus	4 (8.3%)	7 (14.6%)	11 (11.5%)
Hypertension	2 (4.2%)	6 (12.5%)	8 (8.3%)
Smoking	0 (0%)	4 (8.3%)	4 (4.2%)
Alcohol Consumption	0 (0%)	2 (4.2%)	2 (2.1%)
Ischemic Heart Disease	0 (0%)	1 (2.1%)	1 (1%)

Surgical outcomes

Length of Hospital Stay and Delay in Discharge

Table 8 summarizes the distribution of hospital stay durations among the COVID-19 positive and negative cohorts. The results show that COVID-19-positive patients had a mean hospital stay of 13.44 days (SD = 14.29). Specifically, 41 out of 48 patients (85.4%) were hospitalized for 1-20 days, with six out of 48 patients (12.5%) staying for more than 20 days. Conversely, COVID-19-negative patients had a mean stay of 6.63 days (SD = 4.96), with 44 out of 48 patients (91.7%) staying 1-20 days and only three out of 48 patients (6.3%) exceeding 20 days. The observed difference in length of stay between the two groups was statistically significant (P = 0.002), indicating that COVID-19-positive patients had a considerably longer length of hospital stay.

**Table 8:** Distribution of patients according to length of hospital stay (days) P value = 0.002, significant, Student t Test

Length of Hospital Stay (Days), n (%)	COVID-19-Positive (n=48), n (%)	COVID-19-Negative (n=48), n (%)	Total (n=96), n (%)
<1	1( 2.1%)	1 (2.1%)	2 (2.1%)
1-20	41 (85.4%)	44 (91.7%)	85 (88.5%)
>20	6 (12.5%)	3 (6.3%)	9 (9.4%)
Mean ± SD	13.44±14.29	6.63±4.96	10.03±11.18

Table 9 illustrates the distribution of discharge delays among both cohorts. A substantial proportion of COVID-19-positive patients (36 out of 48, 75%) experienced delays in discharge, whereas only six out of 48 (12.5%) COVID-negative patients experienced similar delays. These delays were measured against the institution's standard discharge timelines, demonstrating a notable deviation for COVID-19-positive patients. This difference was highly significant (P ≤ 0.001), indicating that discharge delays were much more prevalent among the COVID-19-positive cohort.

**Table 9:** Distribution of patients accordng to delay in discharge P-value ≤ 0.001, significant, Chi-Square Test

Delay in Discharge	COVID-19-Positive (n=48), n (%)	COVID-19-Negative (n=48), n (%)	TotalL (n=96), n (%)
Present	36 (75%)	6 (12.5%)	35 (36.5%)
Absent	12 (25%)	42 (87.5%)	61 (63.5%)

Postoperative Complications and Additional Findings

As per the medical records reviewed, COVID-19-negative patients did not present with significant complications following surgery, with all patients being discharged in a condition deemed stable according to established emergency care procedures at the hospital of study. In contrast, patients who had concurrent COVID-19 infection during emergency surgeries experienced lengthier hospital stays and a more complex postoperative course. Detailed information on postoperative complications and additional findings in the COVID-19-positive cohort is provided in Table 10.

**Table 10:** Postoperative complications and additional findings in the COVID-19-positive cohort ARDS: acute respiratory distress syndrome, UTI: urinary tract infection

Complications and Additional Findings	Frequency IN COVID-19 Positive Cohort (n = 48), n (%)
Markedly Elevated Postoperative D-Dimer Levels	18 (37.5%)
ICU Admission	8 (16.7%)
Massive Hemorrhage Requiring Blood Transfusion	3 (6.3%)
Spo2 Drop Requiring Endotracheal Intubation	9 (18.7%)
Pulmonary Complications	5 (10.4%)
Progressive Desaturation	1 (2.1%)
Atelectasis	1 (2.1%)
ARDS	3 (6.3%)
Surgical Site Infection	4 (8.3%)
Acute Kidney Injury	3 (6.3%)
Prolonged Intubation	2 (4.2%)
Shock	1 (2.1%)
Cardiac Arrest	1 (2.1%)
Death	3 (6.3%)
Additional Findings:	
Postoperative Prolonged Anticoagulation	36 (75%)
Elevated Postoperative Blood Counts	7 (14.6)
Intraoperative Detection of Notable Large Abdominal Blood Clots	3 (6.3%)
Postoperative Visual Disturbance and Seizure Episode	1 (2.1%)
Postoperative Wound Dehiscence	1 (2.1%)
Postoperative Severe Electrolyte Imbalance	1 (2.1%)
Sick Euthyroid Syndrome	1 (2.1%)
Postoperative UTI	2 (4.2%)

## Discussion

This retrospective matched cohort study provides an in-depth analysis and insights into the effects of COVID-19 infection on emergency surgical interventions and various surgical outcome parameters. The use of a matched cohort design allowed for a robust comparison between COVID-19-positive and COVID-19-negative patients, minimizing selection bias and providing clear insights into how COVID-19 affects surgical recovery. The comprehensive analysis of postoperative findings has contributed valuable data toward understanding the impact of COVID-19 on surgical outcomes.

Throughout the pandemic, elective surgeries were largely put on hold with all surgical departments prioritizing emergency procedures and surgical interventions for cancer patients. Studies have reported that delays in medical care during the pandemic were a major factor in the rise of emergency surgical presentations. For instance, a retrospective analysis of foot and ankle surgical procedures in 2020 revealed that patients with diabetic foot complications experienced significantly increased rates of both minor and major amputations [13]. Additionally, a study on vascular surgery patients in Lombardy during the COVID-19 outbreak reported that patients with COVID-19 had an elevated risk of presenting with acute limb ischemia (ALI) requiring urgent vascular intervention [14].

The data from our study consistently demonstrate that patients with concurrent COVID-19 infection during emergency surgical procedures faced significantly lengthier hospital stays and more challenging postoperative recoveries when compared with those without COVID-19. Additionally, these patients experienced higher rates of ICU admissions, further emphasizing the severity of their postoperative course.

Length of hospital stay

A statistically significant difference in postoperative hospital stay durations was observed between the COVID-19 positive and negative cohorts. Notably, the mean duration of hospitalization for COVID-positive patients was 13.44 days (SD = 14.29), which was substantially longer than the 6.63 days (SD = 4.96) observed for COVID-negative patients (P = 0.002). Moreover, 36 out of 48 COVID-positive patients (75%) experienced delays in discharge compared to just six out of 48 (12.5%) of their COVID-negative counterparts, with a highly significant difference (P ≤ 0.001). These delays were assessed relative to the institution's standard discharge timelines. This evidence is consistent with findings from several studies that have noted prolonged recovery times and extended hospital stays in patients with COVID-19. A retrospective analysis conducted by Pratha et al. reported an average hospital stay of 11.78 days for COVID-19 positive patients, compared to 8.43 days for those without the virus [15]. Furthermore, Rasslan et al. reported a median hospital stay of 30 days for COVID-19 patients with severe respiratory distress, highlighting the extreme impact of the virus on recovery times [9]. A study done on unvaccinated cardiac surgery patients with COVID-19 infection by Ismail et al. also corroborated our findings by demonstrating longer recovery periods for COVID-19 positive patients [16].

Complications and additional postoperative findings

Results from our retrospective analysis illustrate the wide range of complications and postoperative challenges faced by COVID-19-positive patients who underwent emergency surgical procedures amid the pandemic.

D-Dimer Levels

A unique finding in our study that hasn’t been separately addressed in research thus far is the markedly elevated D-dimer levels in COVID-19 patients in the post-operative phase. Normal D-dimer levels usually fall below 0.50 µg/mL or 500ng/mL [17]. While the link between increased D-dimer levels and COVID-19 infection is well-documented, it is also crucial to recognize that surgical stress can further elevate D-dimer levels. In a study conducted by Dindo et al on the kinetics of D-dimer after general surgery, it was seen that D-dimer levels peaked on the seventh postoperative day and then declined exponentially with a daily clearance rate of 6% (half-life of 11 days) [18]. Type I surgeries (non-abdominal) showed normal peak levels, Type II surgeries (intra-abdominal) peaked at 1500 ng/ml and normalized in 25 days, while Type III surgeries (retroperitoneal/liver) peaked at 4000 ng/ml and normalized in 38 days. A study by Kabir et al. investigated D-dimer trends as prognostic indicators for COVID-19 and found that a D-dimer level of ≥2 µg/mL was associated with a higher mortality rate (77%) and increased oxygen requirements [17]. In our study, 18 COVID-19-positive patients (37.5%) exhibited significantly elevated D-dimer levels exceeding 4 µg/mL postoperatively. Additionally, D-dimer levels in this cohort showed fluctuations rather than the exponential decline that is expected one week following surgery. Furthermore, 36 COVID-19-positive patients (75%) required postoperative extended anticoagulation beyond one week, unlike the COVID-19-negative cohort. This underscores the likelihood of adverse postoperative outcomes and heightened mortality in this patient subset.

Postoperative and Intraoperative Complications

Research consistently shows a link between COVID-19 infection in surgical patients and more adverse postoperative outcomes. The most frequently encountered postoperative complications in these studies were respiratory failure, acute respiratory distress syndrome (ARDS), cardiac arrest, sepsis or shock, and acute kidney injury (AKI) [8,19]. Moreover, medical literature indicates a notably high incidence of both pulmonary and thrombotic complications in COVID-19-positive patients. An international multicenter study identified that 32.9% (344 of 1045) of COVID-19 patients undergoing emergency surgery developed pulmonary complications, with rates higher in patients with preoperative respiratory findings [2]. Similarly, an international cohort study conducted by the COVIDSurg Collaborative found that a significant proportion of COVID-19-positive patients developed pulmonary complications following surgery compared to those who were COVID-19-negative [5]. Complementing this evidence, Gulinac et al. highlighted that a significant proportion of COVID-19 patients developed ARDS, along with other pulmonary complications, based on studies reviewed in their article [20]. Additionally, a study by Doglietto et al. in Italy revealed that patients with COVID-19 had notably higher rates of pulmonary and thrombotic complications with odds ratios of 35.63 and 13.2, respectively [6]. Further validation comes from Jonker et al. [21] and Carrier et al. [22], who reported significantly elevated complication rates in patients with COVID-19 (P < 0.01). In alignment with existing literature, our study identified several significant complications in the COVID-19-positive cohort (n = 48). Pulmonary complications were observed in five patients (10.4%) with COVID-19, which included ARDS in three patients (6.3%), progressive desaturation in one patient (2.1%), and atelectasis in one patient (2.1%). Two patients (4.2%) required prolonged intubation during anesthesia, and nine patients (18.7%) experienced significant oxygen saturation (SpO2) drops necessitating endotracheal intubation during the surgical procedure. Other notable complications included massive hemorrhage requiring blood transfusion in three patients (6.3%), surgical site infection in four patients (8.3%), acute kidney injury in three patients (6.3%), shock in one patient (2.1%), and cardiac arrest in one patient (2.1%). Eight (16.7%) COVID-19-positive patients in our study required ICU admission postoperatively, whereas this wasn’t necessary for those without COVID-19 infection. This aligns with prior studies that have reported significantly higher rates of ICU admissions among COVID-19-positive surgical patients and are likely a major contributor to the lengthier hospital stays observed in them [8,23,24].

In addition to the aforementioned complications, additional findings in the COVID-positive cohort include elevated postoperative blood counts in seven patients (14.6%), intraoperative detection of large abdominal blood clots in three patients (6.3%), visual disturbances and a seizure episode in one patient (2.1%), wound dehiscence in one patient (2.1%), severe electrolyte imbalance in one patient (2.1%), sick euthyroid syndrome in one patient (2.1%), and postoperative urinary tract infection (UTI) in two patients (4.2%).

Mortality Rate

We recorded a mortality rate of 6.3% (three cases) among COVID-19-infected patients, which is notably lower than the rates found in several other studies. For instance, Martellucci et al. reported that a quarter of COVID-19 patients experienced mortality, while Doglietto et al. found a rate of 19.51% (eight patients; OR = 9.5; 95%CI, 1.77-96.53) among COVID-19-positive patients, significantly higher than their controls [25,6]. The COVIDSurg Collaborative documented a 30-day mortality rate of 23.8% (268 of 1128) for patients with perioperative SARS-CoV-2 infection [5]. Similarly, Knisely et al. identified a perioperative mortality rate of 16.7% (six out of 36 patients) for COVID-19 patients, contrasted with 1.4% (six out of 432 patients) in non-infected subjects, underlining a notable risk increase [8]. Additionally, Singh et al. reported a staggering 30-day mortality rate of 52% (13 out of 25 patients) among COVID-19 patients undergoing surgery [26]. The relatively lower mortality rate of 6.3% (three cases) observed in our study could be indicative of variations in patient demographics, surgical urgency, and management protocols. These findings collectively underscore the heightened mortality risk faced by surgical patients with COVID-19, emphasizing the critical need for vigilant management and preventive strategies in this vulnerable population.

Limitations and future directions

Despite its contributions, our study is subject to notable limitations. The retrospective design of this study and the relatively small sample size limit the ability to establish causality. The findings of our study are specific to a single hospital setting in India, restricting the generalizability of our results to other regions or health systems. Its reliance on medical records may be subject to inaccuracies or incomplete data entries. Furthermore, medical records showed no detailed entries for the COVID-19-negative cohort, despite documentation of their normal monitoring and timely discharge without complications. The heightened attention and observation of COVID-19 patients, due to the novel and unpredictable nature of the disease, may have resulted in a discrepancy in the level of documentation and potential oversight between the two groups. Additionally, the study also lacks long-term follow-up data, which could provide further insights into the lasting impacts of COVID-19 on surgical recovery.

Future research should focus on prospective studies with large sample sizes to validate these findings and explore the mechanisms behind the observed complications in COVID-19 patients undergoing surgery. Multi-center studies across different geographic locations and healthcare settings could enhance the generalizability of the results. Investigating the impact of vaccination status on surgical outcomes in COVID-19 patients will be crucial for understanding how immunity levels may influence recovery. Additionally, exploring long-term postoperative outcomes and quality-of-life measures will provide a more comprehensive understanding of the enduring effects of COVID-19 on surgical recovery.

## Conclusions

This single-center retrospective analysis demonstrates a significant increase in the length of hospital stays with higher rates of ICU admissions and postoperative complications among COVID-19-positive patients undergoing emergency surgery relative to their COVID-19-negative counterparts. The results highlight crucial implications for clinical practice, emphasizing the importance of preoperative COVID-19 testing and optimized perioperative care to mitigate risks. Future research should focus on conducting larger, multi-center prospective studies to corroborate these observations.
